# Colostrum Lactoferrin Following Active and Recovered SARS-CoV-2 Infections during Pregnancy

**DOI:** 10.3390/biomedicines12051120

**Published:** 2024-05-17

**Authors:** Paulina Gaweł, Błażej Łukianowski, Katarzyna Kościelska-Kasprzak, Dorota Bartoszek, Magdalena Krajewska, Barbara Królak-Olejnik

**Affiliations:** 1 Department of Neonatology, Wroclaw Medical University, 50-556 Wroclaw, Poland; barbara.krolak-olejnik@umw.edu.pl; 2 Department of Pathomorphology and Clinical Cytology, Wroclaw Medical University, 50-556 Wroclaw, Poland; lukianowski.blazej@gmail.com; 3 Department of Nephrology and Transplantation Medicine, Wroclaw Medical University, 50-556 Wroclaw, Poland; katarzyna.koscielska-kasprzak@umw.edu.pl (K.K.-K.); dorota.bartoszek@umw.edu.pl (D.B.); magdalena.krajewska@umw.edu.pl (M.K.)

**Keywords:** COVID-19, SARS-CoV-2, lactoferrin, colostrum, breastfeeding, human milk, immunity

## Abstract

Lactoferrin (Lf), which is particularly abundant in human breast milk during the early stages of lactation, provides protection against a variety of infections, including viral infections, and has demonstrated activity against severe acute respiratory syndrome coronavirus 2 (SARS-CoV-2). The objective of this study was to measure the concentrations of Lf in the colostrum of mothers with active coronavirus disease 2019 (COVID-19) infections during delivery, in mothers with a history of COVID-19 during pregnancy, and in non-infected controls. In this cross-sectional study, colostrum samples from 41 lactating mothers with a confirmed history of SARS-CoV-2 infection (asymptomatic or symptomatic) (both active and past infections) were collected. Twenty-eight colostrum samples collected during the pre-pandemic period served as a control group. An enzyme-linked immunosorbent assay was performed to analyze the Lf concentrations. Concentrations of Lf in the colostrum samples were closely related to virus infection. Colostrum samples from mothers with confirmed SARS-CoV-2 infections contained higher concentrations of lactoferrin compared with samples from mothers from the control group. The highest concentrations of Lf were found in the colostrum samples of mothers with active SARS-CoV-2 infection during delivery when compared with the post-infection and control samples. This observed increase in lactoferrin suggests that it may be an important protective factor for breastfed infants, a finding which was particularly relevant during the pandemic period and remains relevant whenever a breastfeeding mother is infected.

## 1. Introduction

Although coronavirus disease 2019 (COVID-19) is no longer considered a global emergency by the World Health Organization (WHO), it still remains a long-term problem with various risks and consequences, especially among the most vulnerable populations, such as newborn babies. Breastfeeding guidelines have undergone several changes over time. Early in the pandemic, there was reasonable concern about the possibility of mother-to-child transmission of SARS-CoV-2 virus through breast milk; therefore, strict measures, such as separation of the mother–infant dyad or temporary suspension of breastfeeding, were taken. It is now known that the SARS-CoV-2 genome is generally not found in the breast milk of COVID-19-infected women, and that vertical transmission through breast milk seems unlikely [1]. Moreover, as the benefits of breastfeeding outweigh any risk to the baby, breastfeeding was reintroduced as a global recommendation for women with COVID-19, with the caveat that preventive measures should be adhered to [2].

Initial emergency authorizations for new vaccines were made only for children aged 16 years or older. Subsequent authorizations appeared for children gradually; initially for those aged 12 years and older, then 5 years and older. On 17 June 2022, the Food and Drug Administration (FDA) issued emergency authorization for the Moderna COVID-19 vaccine for children aged 6 months to 5 years and the Pfizer-BioNTech COVID-19 vaccine for children aged 6 months to 4 years. However, despite the current eligibility of the youngest population for vaccination, the vaccination coverage for this age group remains low. Many factors contribute to such poor results, including jurisdiction, urbanicity, race, ethnicity, parental concerns about safety and, finally, unequal access to safe and effective vaccines. Data from the Centers for Disease Control and Prevention (CDC) regarding COVID-19 vaccine administration in the United States, showed that, as of 31 December 2022, only 547,089 (9.7%) children aged 6–23 months received ≥ 1 COVID-19 vaccine dose and only 4.5% of them completed the vaccination series [3].

As SARS-CoV-2 vaccines are not intended for individuals under 6 months of age, the main source of newborns’ passive immunity against COVID-19 is breast milk, which, in addition to providing specific SARS-CoV-2 antibodies, is a source of various immune protective factors, including lactoferrin [4,5,6].

Lactoferrin (Lf) is a naturally occurring glycoprotein that is particularly abundant in human milk. Its concentration is dependent on the stage of lactation, and the highest levels are found in colostrum. Lf plays a key role in the innate response to infections, demonstrating broad-spectrum activity against both DNA and RNA viruses [7]. Lf has been reported to display significant effectiveness in blocking SARS-CoV-2 from invading host cells by inhibiting viral binding to the host cell surface in the early phase of virus amplification [8]. One of the most likely mechanisms of this protective effect is competition for binding to heparan sulfate glycosaminoglycans (HSPGs), which are used by many coronaviruses either as receptor determinants or as attachment factors, resulting in a decrease in the accumulation of SARS-CoV-2 on the host cell membrane [8]. In vivo experiments have confirmed that SARS-CoV-2 virus cell entry is mediated by high-affinity interactions between the receptor-binding domain (RBD) of the virus spike glycoprotein and the angiotensin-converting enzyme 2 (ACE2) receptor. Lf is able to bind the ACE2 receptor ectodomain with significantly high affinity and therefore block the initial interaction between virus and host cells [9].

A third possible mechanism is a direct interaction of Lf with the spike (S) protein that promotes the host’s attachment and fusion between the viral and cellular membrane; the membrane (M) protein that plays a central role in virus assembly and morphogenesis; and the envelope (E) protein involved in several aspects of the virus’ life cycle, such as assembly, budding, envelope formation, and pathogenesis [10,11,12].

Since early 2020, nutrients and bioactive compounds have been investigated for potential roles in the prevention and/or adjunctive treatment of COVID-19 symptoms. The milk-derived bioactive protein lactoferrin is one these bioactive compounds, and has shown consistent results both in vitro and in vivo [10,13,14].

As morbidity and mortality from COVID-19 are rare among infants, Lf, which is provided via breast milk, has been proposed as a protective factor [15]. Therefore, in this cross-sectional study, we investigated whether Lf concentrations in the colostrum of COVID-19-infected mothers, lactating women with past COVID-19 infection, and healthy controls differed, and whether or not the Lf concentration might have a significant impact on the immune protection of breastfeeding infants. Identifying Lf as another significant defense factor in human breast milk against SARS-CoV-2 may contribute to the promotion of breastfeeding and improvements in lactation rates.

## 2. Materials and Methods

### 2.1. Research Design

Our prospective, cross-sectional, and observational study involved lactating mothers with a history of COVID-19 during their pregnancy, lactating mothers with an active COVID-19 infection during delivery, and pre-pandemic healthy controls. This study design was chosen because it allowed us to observe the outcome of interest (concentration of colostrum lactoferrin) in a group of participants selected for the exposure of interest (virus infection). This study was approved by the Commission of Bioethics at Wroclaw Medical University (Poland)—agreement No. KB-338/2021. All methods were performed in accordance with the Helsinki Declaration.

### 2.2. Setting

Breastfeeding and other methods of feeding human milk to infants are highly recommended after birth. Poland has a high rate of initiating breastfeeding after birth (97%) and a rapid abandonment of exclusive breastfeeding (43.5% at 2 months, 28.9% at 4 months, and 4% at 6 months) [16]. Our study was conducted at the Wroclaw University Hospital during the third wave of the pandemic (15 February 2021 to 1 May 2021). At that time, the percentage of infants who received any mother’s milk was estimated at 84.5%. Moreover, 83.3% of mothers practiced skin-to-skin contact and roomed with their babies [17].

### 2.3. Study Population

**Figure 1:**
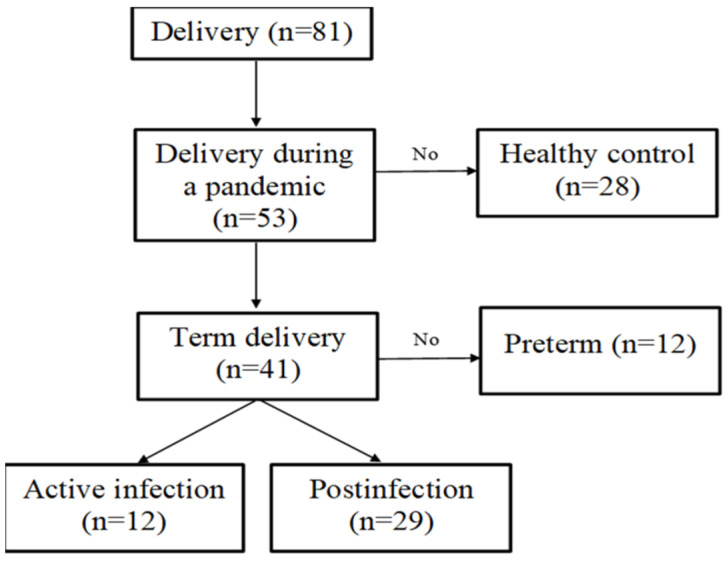
Flowchart of the study group recruitment steps.

A total of 69 women were included in this study (Figure 1). The participating women were patients at the Wroclaw University Hospital, and the inclusion criteria were as follows:

- Quantitative reverse transcription polymerase chain reaction (RT-qPCR)-confirmed coronavirus infection during pregnancy/delivery or being never infected with the virus.
- Lactation in the postpartum period.

Participants were excluded if they were:

- vaccinated against SARS-CoV-2 at the time of the study.
- or delivered preterm.

Mothers collected up to 5 mL of their colostrum between the 3rd and 7th day post partum in the morning hours (8 a.m. to 11 a.m.). According to standard criteria, “colostrum” was classified as the milk collected in the first 7 days after delivery [18]. The needs and best interests of the newborns were always prioritized. All mothers were instructed to express colostrum immediately after feeding the baby, either into sterile plastic containers using clean electric breast pumps or by self-pumping after hand sanitization. The colostrum samples were separated into aliquots, frozen, and stored at −80 °C until the enzyme-linked immunosorbent assay (ELISA) measurements. Control samples came from the pre-pandemic period (second half of 2019) and were collected using exactly the same method and at the same time points as the other groups’ samples. The control group was hospitalized for reasons unrelated to infectious illness, and only for the purpose of giving birth.

### 2.4. Measurements

The concentrations of Lf in the colostrum samples were measured using quantitative enzyme-linked immunosorbent assays (ab200015—Human Lactoferrin SimpleStep ELISA^®^ Kit, Abcam, Cambridge, UK). The colostrum samples had been previously defatted by centrifuging at 500× *g* for 15 min at 4 °C. The aqueous fraction was recentrifuged at 3000× *g* for 15 min at 4 °C, and the final aqueous fraction was collected. The defatted colostrum samples were diluted 10,000,000 times using the sample diluent reagent provided in the kit. The mean coefficients of variation (CVs) of intra-assay and inter-assay precision were CV% = 5.1% and CV% = 5.4%, respectively. The units used for the Lf concentrations were mg/mL of milk.

### 2.5. Data Collection

Recruitment and sample collection were conducted from 15 February to 1 May 2021, during the third wave of the COVID-19 pandemic. All participants provided oral and written consent before any study procedures were performed. Data regarding mothers and their infants were collected at the time of enrollment by filling in a personal questionnaire. All scientific information collected as part of this study was treated confidentially. A unique code was assigned to each study participant to ensure anonymity, and data were stored safely at the study site.

### 2.6. Data Analysis

Descriptive statistics of the studied groups are presented as medians with interquartile ranges (IQRs). The distributions of the studied variables were checked using the Shapiro–Wilk test. All variables presented were shown to be abnormally distributed. The Mann–Whitney U test was used to compare the values of the variables between the groups. The Mann–Whitney U test is a useful statistical tool for comparing two independent groups, especially when they do not meet the assumptions of parametric tests. Its independence from data distribution, resistance to outliers and universality make it often used in scientific research and data analysis. It can be successfully used to compare different types of data, such as rank and ordinal data, which makes it useful in many fields of science. Despite its nonparametric nature, the Mann–Whitney U test demonstrates fairly good statistical power, especially when differences between groups are significant. The Kruskal–Wallis test was used to compare a quantitative variable in more than two groups. The results with *p* < 0.05 were considered significant. Statistical analysis was performed using the STATISTICA 13 statistical package (TIBCO Software Inc., Palo Alto, CA, USA).

## 3. Results

### 3.1. Basic Information Describing Maternal–Infant Dyads

This study involved 69 women, of whom 29 had a history of COVID-19 during pregnancy (8—first trimester; 2—second trimester; and 19—third trimester); 12 had an active infection during delivery; and 28 were pre-pandemic, non-infected controls. Among the study group (total of 41 women), only two women (2.90%) were asymptomatic. Thirty-seven (53.62%) of the women manifested mild to moderate symptoms, and one (1.45%) had severe infection symptoms. The most commonly reported symptoms were fatigue (63.2%), rhinitis (55.3%), cough and loss of taste and smell (47.4%), fever (42.1%), headache (44.7%), muscle ache (36.8%), dyspnea (26.3%), shivers (21.1%), sore throat (18.4%), diarrhea (5.3%), and vomiting (2.6%). The median age of mothers in the study group was 32 years old. The mode of delivery was mostly cesarean section, performed in approximately 53% of all births. The median gestational age at delivery was 40 weeks. No positive SARS-CoV-2 test results were registered among infants born to COVID-19-infected mothers. More than half of the newborns (60.87%) were assigned male. The mean weight of the newborns was 3566 g, while the mean length was 53 cm. Additional characteristics of the study participants and their infants are presented in Table 1.

### 3.2. Lactoferrin Concentrations in Maternal Colostrum Samples

Overall, a total of 81 colostrum samples were collected from all mothers. However, in light of the general knowledge of the higher concentrations of Lf in preterm colostrum [19,20], confirmed by the results from our study (higher concentrations of Lf in preterm colostrum, mean 52.82, range 32.08–77.57, compared with the full-term colostrum, mean 40.17, range 29.26–56.03), only samples collected from mothers who delivered at term (≥37 weeks of pregnancy) were included in the statistical analysis (*n* = 69). Among the other research results, the colostrum of mothers of low-birth-weight newborns (<2500 g) contained the highest concentrations of lactoferrin. The lowest concentrations of lactoferrin were observed in the colostrum of mothers of newborns with fetal macrosomia (>4200 g). Our results are in accordance with other previously conducted studies [21,22].

**Figure 2:**
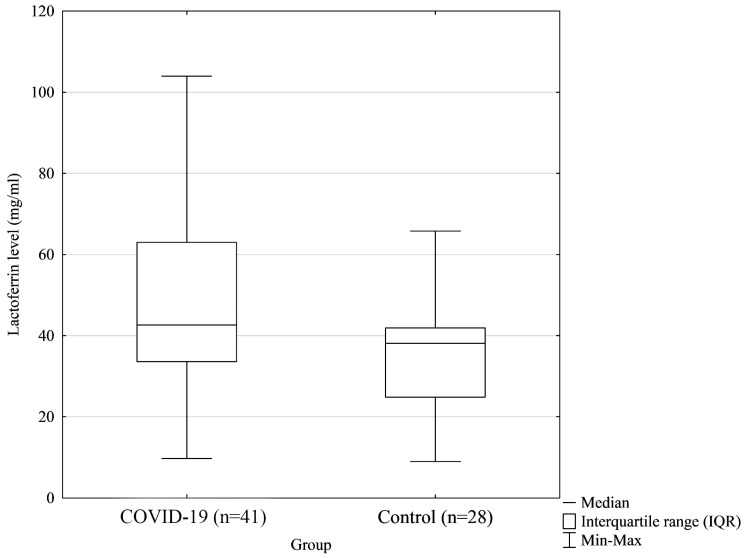
The differences between the median concentrations of lactoferrin in colostrum showed statistical significance (*p* = 0.018).

**Figure 3:**
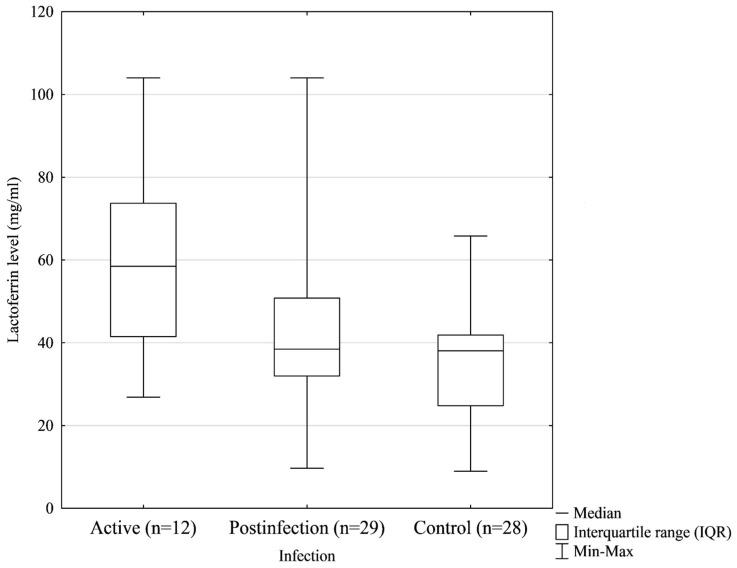
The lactoferrin content in the colostrum samples was dependent on the history of COVID-19 infection. Statistically significant differences were observed (*p* < 0.05).

The concentrations of Lf in the colostrum samples were significantly related to virus infection. Colostrum samples from mothers with confirmed SARS-CoV-2 infection contained higher concentrations of Lf compared with the colostrum samples of mothers from the control group (*p* < 0.018, Figure 2): median 45.71, IQR 33.57–65.70 vs. median 38.08, IQR 24.82–41.91, respectively. Moreover, after dividing the study group into subgroups, we noticed that the highest concentration of Lf was observed in the colostrum of mothers with active SARS-CoV-2 infection during delivery (median 58.50, IQR 41.50–75.18) compared with the post-infection samples (median 40.86, IQR 31.97–56.03) and control samples (median 38.08, IQR 24.82–41.91). The difference was considered statistically significant (*p* = 0.010) (Figure 3). Lf concentrations in the colostrum of mothers with active COVID-19 infection were found to be statistically different compared with those in the colostrum of the control group (Table 2).

Colostrum samples from SARS-CoV-2-positive mothers who developed symptoms (*n* = 37) did not differ significantly in lactoferrin concentration compared with asymptomatic mothers (*p* = 0.843) or healthy mothers (*p* = 0.058) (Table 3). Colostrum samples were collected from mothers during or after SARS-CoV-2 infection. The longest period between the onset of symptoms and the collection of a sample of colostrum was 229 days, the minimum was 3 days, and the average was 81.44 days. The highest mean value of time between the confirmation of infection and collection of the colostrum sample was observed in the group of symptomatic mothers (Table 3).

**Figure 4:**
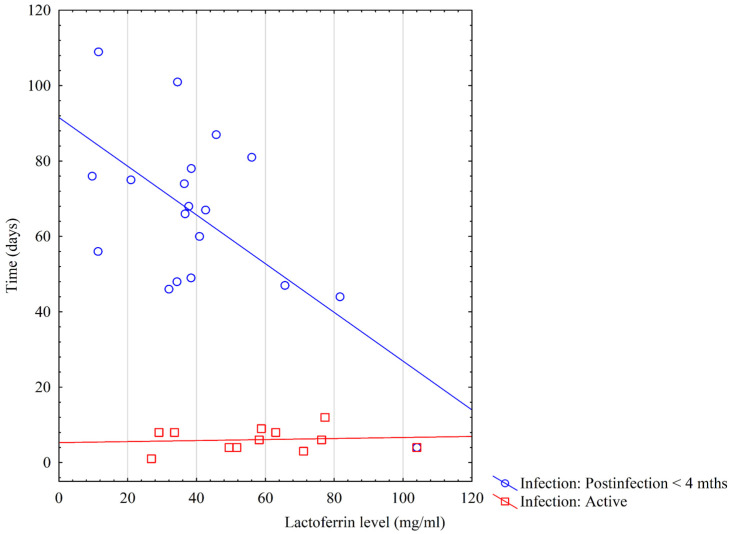
Lactoferrin concentrations in colostrum versus time since confirmed COVID-19 infection.

The analysis below examines the associations between concentrations of Lf in colostrum and the time passed since the mother’s confirmation of COVID-19 infection. Due to the increased risk of secondary SARS-CoV-2 infection over time, we excluded mothers who were more than four months post infection. Among mothers with an active COVID-19 infection, the LF concentration remained constant, while in recovered mothers, a decrease in concentration was observed over time (Figure 4).

## 4. Discussion

In the context of the COVID-19 pandemic, lactoferrin became a subject of increased interest, as it was reported to demonstrate substantial antiviral activity against SARS-CoV-2 [14]. This antiviral activity may be due to Lf’s ability to block the cellular attachment and further replication of the coronavirus; its immunomodulatory activity, which up- and downregulates the expression of innate and adaptive immune cells; its contribution to the homeostasis of mucosal surfaces; and its ability to sequester free iron, protecting against insult-induced oxidative stress and the subsequent “cytokine storm” associated with severe COVID-19 infection [23]. For these reasons, various studies have, since the outbreak of the coronavirus, evaluated the efficacy of external supplementation with lactoferrin as a preventive, adjunctive, or curative therapy [24,25,26]. In a study by Serrano et al., performed on a group of 75 patients with symptomatic COVID-19, liposomal bovine lactoferrin (bLf) was administered in a dose ranging from 256 to 384 mg/day. As a result, rapid recovery within the first 4–5 days was observed in all patients (100%), and a reduced severity of symptoms associated with COVID-19 was also observed. Additionally, lower doses of Lf (128–192 mg/day) received by family members who had contact with infected patients (256 persons) have been shown to prevent viral infection [25].

Additionally, combined use of Lf and vitamin D as an adjuvant for COVID-19 management was suggested. Vitamin D plays a crucial role in promoting the immune response. Due to its anti-inflammatory and immunoregulatory properties, vitamin D is essential for activating the immune system’s defenses and improving immune cell function. Beyond Lf activity against the SARS-CoV-2 virus, recent studies have demonstrated that lactoferrin is a potential activator of the vitamin D receptor. Synergistic action of both compounds might be a valuable tool with which to prevent the spread and worsening of the infection [13].

Breastfeeding gives newborns and infants an opportunity to acquire immune protection. However, little is known about the influence of the SARS-CoV-2 virus on breast milk compounds, in particular concerning the possible protective factors in breast milk. There are only a small number of studies which have addressed this issue. In one study, conducted by Guo et al., total protein amounts in the COVID-19 colostrum group were significantly higher (4.1 times) than in the control colostrum group; whereas casein proteins exhibited significantly lower abundances (3.9 times lower) and whey proteins, with their immune-related activity against SARS-CoV-2, were 7.2 times higher [27]. COVID-19 was also reported to reduce the concentrations of Fe, Cu, Se, Ni, V, and aluminum (Al) and increase Zn when compared with pre-pandemic control samples [28]. 

In terms of the endogenous lactoferrin present in breast milk, which is a key contributor to newborns’ innate immunity, there is still a knowledge gap. To verify whether Lf might be a significant protective factor during a viral epidemic and to provide vital protection to breastfeeding newborns, we first wanted to determine whether there is any relationship between SARS-CoV-2 infection and Lf concentrations in the colostrum, as this relationship has been poorly investigated to date. In the present study, an increase in the concentration of Lf during COVID-19 infection was observed, which provides a basis for further research regarding the influence and effectiveness of Lf in protecting breastfed infants from viral infections.

Our analysis showed that Lf concentration in the colostrum is influenced by the mother’s history of COVID-19 infections during pregnancy and delivery. Higher colostrum Lf concentrations were found in the study group, and, notably, the highest concentrations were observed in the subgroup of women with an active infection during delivery. The elevated concentrations of Lf in patients infected with SARS-CoV-2 might be explained by the study of Reghunathan et al., conducted in 2005. In that study, researchers found that several genes are highly upregulated during coronavirus infection. The expression of genes coding for Lf showed the largest increase in SARS patients, with a fold-change of almost 150 when determined via microarray assay and that of 92.6 when verified via real-time PCR analysis [29]. The upregulation of endogenous Lf production may result in higher amounts of Lf being transmitted through breast milk.

Only one previous study, conducted by Briana et al., has evaluated the concentration of lactoferrin in the colostrum of SARS-CoV-2-positive mothers. Among the study group only nine mothers tested positive for SARS-CoV-2 RNA upon admission for delivery, while four had a history of precedential infection during pregnancy. In contrast with our study, the researchers found no difference in the Lf concentration between SARS-CoV-2-positive mothers and controls. However, their study size was small—only 13 mothers in the study group and 15 in the control group [30].

As the main mechanism of Lf’s antiviral activity against SARS-CoV-2 is binding to HSPG receptors [8], in severely infected mothers, lower concentrations of Lf in external fluids (i.e., breast milk) may be related to a “compensatory decrease” due to a significant rise in the blocking of the site of cellular attachment of SARS-CoV-2; however, this is only hypothetical. Our data show a different colostrum Lf ‘profile’ regarding the severity of infection, with lower Lf concentrations found in the colostrum of symptomatic mothers. However, our findings for this subgroup analysis were not statistically significant. In the study of Briana et al., similarly, the subgroup of symptomatic mothers (*n* = 7) presented lower colostrum Lf concentrations when compared with controls and asymptomatic mothers [30]. In both studies, the small sample size of asymptomatic mothers could have influenced the analysis, and therefore, our hypothesis should be further examined on larger groups.

Among other studies concentrating on the association between a maternal history of COVID-19 infection and the lactoferrin concentrations in the colostrum, an inverse relationship was found in a prospective study by Turin et al. That study involved 346 mothers of low-birth-weight newborns and showed that maternal peripartum infection was significantly associated with lower Lf concentrations in the colostrum [31]. Lönnerdal et al. showed similar results, with higher Lf concentrations being found in healthy Peruvian mothers compared with ill mothers (the illnesses included urinary tract infection, chorioamnionitis, and respiratory or skin infections) [32]. In another cross-sectional study, by Fujita et al., of a group of 200 mothers in northern Kenya, the link between milk lactoferrin and maternal inflammation was evaluated. The results of that study suggest that mothers produce more Lf when they experience inflammation [33].

The first and most important limitation of this study is its small sample size. Although the statistical analysis showed strong associations that are assumed to be sufficient to answer the research question and though the obtained results give an overview of the problem addressed, there is still a need to verify these results on a larger number of participants. The next limitation is the long storage time of pre-pandemic control samples, which may have affected the lactoferrin concentrations in these colostrum samples. However, it would be impossible to gather a concurrent control group that has never been exposed to the virus.

## 5. Conclusions

In conclusion, breast milk, especially the colostrum, is the best source of innate immunity for growing newborns. The benefits of breastfeeding are widely known and, even in the context of the pandemic, are considered to far outweigh the risks of infection. The concentration of lactoferrin in the colostrum during and after SARS-CoV-2 infection increases, suggesting that, beyond specific antibodies, lactoferrin may be another important protective factor for breastfed infants, which is particularly relevant during the pandemic period.

## Figures and Tables

**Table 1:** Characteristics of study participants and their neonates.

	Study Group *n* = 41		Control Group *n* = 28	*p* Value
	Active Infection *n* = 12	Post Infection *n* = 29		
Age (years)	31, 28–33	32, 30–36	30, 28–32	0.048
Gestational age at birth (weeks)	39, 39–40	40, 38–41	40, 39–40	0.240
Mode of delivery:				0.030
Vaginal	3 (25.00%)	11 (37.93%)	18 (64.29%)	
Cesarean section	9 (75.00%)	18 (62.07%)	9 (32.14%)	
Missing data	0	0	1 (3.57%)	
Neonates’ assigned sex:				0.500
Female	6 (50.00%)	9 (31.03%)	11 (39.29%)	
Male	6 (50.00%)	20 (68.97%)	16 (57.14%)	
Missing data	0	0	1 (3.57%)	
Neonates’ weight (g)	3365, 3220–3425	3630, 3440–3800	3730, 3395–3880	0.100
Neonates’ length (cm)	52, 51–53	53, 52–55	54, 52–57	0.013

Data are presented as *n* (%) or median with interquartile range (IQR). The *p* value < 0.05 was considered statistically significant.

**Table 2:** Lactoferrin concentrations in colostrum by the history of infection.

	*n*	Lactoferrin Concentrations (mg/mL)
Total COVID-19	41	45, 33–65
Active COVID-19	12	58, 41–75
Post infection COVID-19	29	40, 31–56
Control	28	38, 24–41
		**Lactoferrin Concentrations** ***p*-Value**
Total COVID-19 vs. control		0.018
Active COVID-19 vs. control		0.001
Post infection COVID-19 vs. control		0.160
Active COVID-19 vs. post infection COVID-19		0.080

Data are presented as n or median with interquartile range (IQR). The *p* value < 0.05 was considered statistically significant.

**Table 3:** Lactoferrin concentrations and time since COVID-19 infection in the subgroups of mothers participating in the study.

	*n*	Lactoferrin Concentrations (mg/mL)	Time (Days)
Total COVID-19	41	42, 33–62	66, 8–109
Symptomatic COVID-19	37	42, 33–62	67, 12–157
Asymptomatic COVID-19	4	43, 31–77	6, 4–54
Control	28	38, 24–41	0
		**Lactoferrin Concentrations** ***p*-Value**	
Total COVID-19 vs. control		0.018	
Symptomatic COVID-19 vs. control		0.058	
Asymptomatic COVID-19 vs. control		0.319	
Symptomatic COVID-19 vs. asymptomatic COVID-19		0.843	

Data are presented as *n* (%) or median with interquartile range (IQR). The *p* value < 0.05 was considered statistically significant.

## Data Availability

The data presented in this study are available upon reasonable request from the corresponding author.
